# Comparison of the sagittal sinus cross-sectional area between patients with multiple sclerosis, hydrocephalus, intracranial hypertension and spontaneous intracranial hypotension: a surrogate marker of venous transmural pressure?

**DOI:** 10.1186/s12987-017-0066-1

**Published:** 2017-07-06

**Authors:** Grant A. Bateman, Jeannette Lechner-Scott, Ross Copping, Christopher Moeskops, Swee Leong Yap

**Affiliations:** 1 Department of Medical Imaging, John Hunter Hospital, Locked Bag 1, Newcastle Region Mail Center, Newcastle, 2310 Australia; 2 Newcastle University Faculty of Health, Callaghan Campus Newcastle, Newcastle, Australia; 3 Department of Neurology, John Hunter Hospital, Newcastle, Australia; 4 Hunter Medical Research Institute, Newcastle, Australia

**Keywords:** Multiple sclerosis, Hydrocephalus, Idiopathic intracranial hypertension, Spontaneous intracranial hypotension

## Abstract

There is evidence that patients with multiple sclerosis (MS) and hydrocephalus share some common pathophysiological mechanisms. Alterations in CSF pressure are known to affect cerebral venous sinus geometry. To further explore these mechanisms, we measured the superior sagittal sinus (SSS) cross-sectional area 3 cm above the torcular using T2 images in 20 MS, 10 spontaneous intracranial hypotension (SIH), 21 hydrocephalus and 20 idiopathic intracranial hypertension (IIH) patients and compared with 20 matched controls. The SSS area was reduced by 25% in hydrocephalus (p = 0.0008), increased by 22% (p = 0.037) in SIH and unchanged in IIH compared to matched controls. In MS there was a 16% increase in SSS area (p = 0.01).The findings suggest that changes in SSS cross-sectional are common between MS and SIH patients, while in hydrocephalus and IIH these are different.

**Electronic supplementary material:**

The online version of this article (doi:10.1186/s12987-017-0066-1) contains supplementary material, which is available to authorized users.

## Background

In a recent paper by the current authors, MS was shown to be similar to normal pressure hydrocephalus (NPH) with regards to vascular compliance and pulsation propagation [1]. The correlation between MS and NPH could suggest that there may be a CSF absorption abnormality in MS similar to NPH. The walls of the sagittal sinus have been shown to move depending on changes in the transmural pressure. In a single case of chronic hydrocephalus, the sagittal sinus cross-sectional area was reduced [2], in 16 IIH patients the sagittal sinus cross-sectional area was normal [3] and in spontaneous intracranial hypotension the sinuses are qualitatively dilated (although this has never been measured quantitatively) [4]. Therefore, it is suggested the size of the sagittal sinus cross-section could be used as a surrogate measure of the transmural pressure. The purposes of the current study are (1) to better document the change in sinus size which occurs in hydrocephalus, IIH and SIH and (2) to document the size of the sinus in a cohort of MS patients to see which disease MS most closely resembles.

## Methods

### Subjects

In a previous study into hydrocephalus undertaken by one of the authors, there were 21 patients with chronic hydrocephalus, mean age 45 ± 10 years, 7 female and 14 male. There were 20 controls, mean age 44 ± 10 years with 8 females and 12 males [2]. These patients were entered into the current study, and the detailed clinical data for these patients can be reviewed in the prior publication [2]. Twenty patients with idiopathic intracranial hypertension were entered from a prior publication undertaken by one of the current authors, there were 18 females and two males of average age 40 ± 11 years [5]. In all 20, there was an increase in CSF opening pressure above 18 mmHg. There was a normal ventricular size and no apparent cause for the pressure rise in these patients. The radiology department information system was searched for patients undergoing an MRI examination over the last decade who had a final diagnosis of spontaneous intracranial hypotension. There were 6 females and 4 males of average age 46 ± 11 years. All patients had documented CSF pressures below 4.4 mmHg with CSF leaks in the spinal canal or skull base. In a previous study by the current authors, twenty consecutive relapsing remitting MS patients underwent an MRI examination, all were female [1]. The demographic and clinical details are available in the previous publication [1].

### MR and analysis

All patients were imaged on a 1.5 T superconducting magnet (Vario; Siemens, Erlangen Germany). In all patients, a standard brain MRI consisting of, T1 sagittal, T2 axial and diffusion weighted axial images was performed. The axial images were angled to be parallel to the skull base. In the MS patients an additional 3D T1 post contrast series was acquired (this was originally used to detect lesion enhancement). The MRI imaging was sourced from the hospital picture archiving and communication system (PACS) and therefore all measurements were performed on the original data. In all patients and controls, a T2 axial image was selected which passed through the superior sagittal sinus 3 cm above the torcular. The area and circumference of the flow void in each sinus was measured by one of the authors (GB) using the scanner’s measurement tool. In addition the MS patients had an angulated axial slice reconstructed perpendicular to the long axis of the sagittal sinus 3 cm above the Torcular, from the post contrast 3D images. Using the contrast outlined lumen of the sinus, the cross-sectional area and circumference was measured independently by 4 of the authors (GB, RC, CM, SLY). An average of each of these measurements was calculated. The circularity of all of the sinuses was calculated using the formula:

$${\text{C}}=4{\uppi}{\text{A/L}}^{{\text{2}}}$$

where A is the area and L the circumference.

### Statistical analysis

Group means and standard deviations were obtained for each of the measurements. In the contrast study, the MS patients had an intraclass correlation coefficient calculated. A non-paired T test, with a p value of less than 0.05 was used to indicate statistical significance.

## Results

The raw data supporting the findings of this study can be found in Additional file 1. The data is summarized in Table 1.

**Table 1:** Sinus cross section area and circumference

	Age	Sinus area	Circumference	Circularity
	Years	mm^2^	mm	
Control n = 20				
Mean	44	42.1	27.5	0.70
SD	10	10.0	3.8	0.11
IIH n = 20				
Mean	41	42.4	26.4	0.76
SD	11	11.9	3.9	0.11
p value Con vs IIH	0.33	0.94	0.34	0.07
Hydrocephalus n = 21				
Mean	45	31.4	23.0	0.73
SD	10	8.4	3.2	0.09
p value Con vs HY	0.82	0.0008*	0.0002*	0.29
SI Hypotension n = 10				
Mean	46	51.3	28.9	0.76
SD	11	12.7	2.8	0.07
p value Con vs SIH	0.63	0.037*	0.33	0.11
MS n = 20				
Mean	45	49.0	28.8	0.75
SD	12	6.5	2.8	0.12
p value Con vs MS	0.99	0.01*	0.23	0.14

*Con* control, *HY* hydrocephalus, *IIH* idiopathic intracranial hypertension, *mm* ^2^ mm squared, *mm* millimeters, *MS* multiple sclerosis, *SD* standard deviation, *SIH* spontaneous intracranial hypotension

* t test p value <0.05

In IIH, the sinus area, circumference and circularity were not significantly different to the controls. In hydrocephalus, the sinus area was reduced by 25% and the circumference by 16% (p = 0.0008 and 0.0002 respectively). In SIH the sinus area was increased by 22% (p = 0.037). The MS sinus cross-sectional area was 16% larger than controls (p = 0.01), the circumference and circularity were not significantly different. The averaged 3D T1 data in the MS patients was not significantly different to the T2 data with the area being 48.6 mm^2^ compared to 49.0 mm^2^. The overall intraclass correlation coefficient for the four independent measurements was 0.87.

## Discussion

The initial portion of the study was undertaken using T2 images. However, the T2 images were angled to the skull base and are thus not exactly perpendicular to the sinus. Unlike the T2 images, the 3D T1 images are able to be reconstructed perpendicular to the sinus. Measurements of both the T2 and post contrast T1 images in MS patients were performed to test for possible inaccuracies due to distortion in the T2 imaging and to gauge the inter observer reliability. There was no significant difference between the two methods with good inter observer reliability. The cross-sectional area in the controls i.e. 42.1 mm^2^ compares favourably with recently published reference data showing an area of 43 mm^2^ three cm from the torcular [6]. The shape of the sinuses was tested by calculating the circularity. Circularity is defined by the formula $4\uppi{\text{A}}/{\text{L}}^{2}$ where A is the cross-sectional area in mm^2^ and L the circumference in mm [7]. It can be seen that a circle of radius R would return a circularity value of 1. An equilateral triangle of side length 1 unit would return a value of 0.6. We can see from Table 1 that the shape of the sinuses of all groups was midway between that of a circle and triangle and not significantly different to each other. The sagittal sinus circularity has been previously published in control subjects and is 0.77 [7], which is not significantly different to the current study.

The cerebral sinuses lie between the fibrous dura mater and the endosteum. The sagittal sinus consists of a venous channel passing through a split in the dura as it passes from the falx cerebri to the skull [8]. The dura at the base of the sinus is attached to the endosteum of the skull and is fixed. The other two walls of the sinus are attached to the falx cerebri and are relatively fixed at this point. Between the three fixed vertices, the two walls separating the subarachnoid space from the sinus lumen are free to move. In the transverse sinuses the free walls have been noted to be concave, straight or convex [4]. The dural walls of the sagittal sinus are made up of collagen fibres interspersed with fibroblasts and elastin in a ground matrix [9]. Therefore, the walls are expected to have viscoelastic properties [10]. In human dura, small changes in stress (the applied force) directed along dura strips produces corresponding small changes in strain (the percentage change in length), in a linear relationship which follows Hooke’s law [11]. The stretch is recoverable in an elastic fashion provided the elastic limit is not reached. The gradient of the stress vs strain graph is called the Young’s modulus [11]. The Young’s modulus for human dura adjacent to the sagittal sinus in adults was measured at 61 MPa with the dura remaining elastic up to a 20% stretch [10]. Therefore, the amount of stretch induced in the free walls of the sinus is expected to be proportional to the applied transmural pressure gradient provided the tensile strength of the wall remains a constant. Therefore a large transmural pressure should give more stretch and a lower pressure less stretch. However, the area change produced in the vessel by a change in wall length may not be a linear function because of the triangular shape and constraint of the sinus.

### Correlation between transmural pressure and sinus size

The normal transmural venous pressure from CSF to sinus lumen is 4 mmHg [12]. In hydrocephalus patients the CSF outflow resistance is said to be elevated [2]. Therefore, the expected transmural pressure in chronic hydrocephalus should be higher than normal. It follows the wall deflection in hydrocephalus should be larger and cross-sectional area lower than in the control group. From Table 1 we can see that the cross-sectional area of the sinus in hydrocephalus is 25% smaller than the matched controls.

In idiopathic intracranial hypertension there is an elevation in ICP above 18 mmHg, which is not related to an intracranial mass, meningeal process or cerebral thrombosis [13]. Pickard et al. simultaneously measured the ICP and sagittal sinus pressure in 9 patients with IIH and found the transmural sinus pressure to be 1.8 mmHg [14]. King et al. simultaneously measured the CSF pressure at C2 and the SSS pressure in 21 IIH patients and found a gradient of 5 mmHg [15]. These studies are somewhat divergent but added together suggest the transmural pressure may be normal in IIH. In the current study there was no significant difference in sinus size, perhaps correlating with the normal pressure gradient. Similarly, Rohr et al. found no significant difference in the cross-sectional area of the sagittal sinus measured from T2 images 1 cm above the Torcular when comparing IIH with controls [3].

Spontaneous intracranial hypotension (SIH) patients present with positional headaches which are worse in the upright position. The cause is typically a CSF leakage, most frequently within the spinal canal. An opening pressure of less than 4.4 mmHg is diagnostic for SIH [16]. In SIH there is enlargement of the venous sinuses [4] and also dural thickening [17]. In the current cohort, an overall 22% increase in the size of the sinus compared to normal was found. Extensive dural thickening was also found on other imaging (not shown). Given the normal sinus pressure is 7.5 mmHg at age 45 years [2], an opening pressure of 4.4 mmHg would give a reversed pressure gradient accounting for the venous dilatation.

The cross-sectional area of the sinuses in MS is 16% larger than the controls. This is unlike either hydrocephalus or IIH but closest to SIH. The increase in size could favour a decrease in transmural pressure, like SIH. However, this would need to be due to an increase in venous pressure rather than a decrease in ICP. The notion that there is an elevation in venous pressure in MS has been extensively investigated, reviewed and disputed [18–20]. Therefore the cause for the dilatation is undefined and awaits further study.

The dilatation of the sinus walls in MS is similar to the findings of a whole brain 3T susceptibility-weighted study into MS which showed that although the intralesional parenchymal veins were 24% smaller, the extralesional veins in the normal appearing white matter were 20% larger by diameter [21]. This raises another paradox. The compliance of the venous system in MS was noted to be reduced by half when compared to controls [1]. Compliance is the change in volume divided by the change in pressure which brings this about or ${\text{C}}=\Delta{\text{V}}/\Delta{\text{P}}$. Note the change in volume is in absolute terms not relative. If the change in pressure across the venous wall and the tensile strength of the veins were constant, a much larger venous volume in MS should bring about a much larger $\Delta{\text{V}}$. Therefore, the current findings suggest the venous compliance in MS should be larger not smaller. One possible solution to this problem would be if the wall strength were much larger than normal. Coen et al. noted that a change in the type 1 collagen component of the jugular veins occurs in MS perhaps [22] correlating with a possible change in wall strength but this would require further enquiry to ascertain.

### Limitations

The estimation of the cross-sectional area is likely to be more accurate using the post contrast images compared to the T2 images. Unfortunately, post contrast imaging was only available in the MS patients. The wall of the sinus being fibrous is of low signal on T2, similar to the flowing blood, so it is possible part of the wall may be added to the lumen in T2 based cross-sectional area studies. Despite this, the difference between both techniques was found to be negligible. In addition, the plane of the T2 studies is not exactly perpendicular to the direction of flow of the sagittal sinus. A review of all cases showed the average angle of the T2 slices directed away from perpendicular was 8.8° ± 6.2° which would affect the measurement of the antero-posterior dimension of the sinus by the cosine of the angle giving a 1.2% error. The transverse dimension would be unaffected.

## Conclusions

The size of the sagittal sinus cross-section appears to be dependent on the transmural pressure and the tensile strength of the sinus wall. The increase in size of the sinus seen in MS is similar to SIH but the cause of this awaits further study.

## Additional file

**Additional file 1.** Area and circumference raw data.

## Abbreviations

ICP: intracranial pressure
IIH: idiopathic intracranial hypertension
mmHg: millimeters of mercury
MS: multiple sclerosis
NPH: normal pressure hydrocephalus
SIH: spontaneous intracranial hypotension
SSS: superior sagittal sinus
SD: standard deviation
